# Cortical Thickness in Migraine: A Coordinate-Based Meta-Analysis

**DOI:** 10.3389/fnins.2020.600423

**Published:** 2021-01-06

**Authors:** LiQin Sheng, HaiRong Ma, YuanYuan Shi, ZhenYu Dai, JianGuo Zhong, Fei Chen, PingLei Pan

**Affiliations:** ^1^Department of Neurology, Kunshan Hospital of Traditional Chinese Medicine, Suzhou, China; ^2^Department of Central Laboratory, School of Medicine, Affiliated Yancheng Hospital, Southeast University, Yancheng, China; ^3^Department of Radiology, School of Medicine, Affiliated Yancheng Hospital, Southeast University, Yancheng, China; ^4^Department of Neurology, School of Medicine, Affiliated Yancheng Hospital, Southeast University, Yancheng, China

**Keywords:** migraine, cortical thickness, coordinate-based meta-analysis, seed-based d mapping with permutation of subject images, surface-based morphometry

## Abstract

Cortical thickness (CTh) via surface-based morphometry analysis is a popular method to characterize brain morphometry. Many studies have been performed to investigate CTh abnormalities in migraine. However, the results from these studies were not consistent and even conflicting. These divergent results hinder us to obtain a clear picture of brain morphometry regarding CTh alterations in migraine. Coordinate-based meta-analysis (CBMA) is a promising technique to quantitatively pool individual neuroimaging studies to identify consistent brain areas involved. Electronic databases (PubMed, EMBASE, Web of Science, China National Knowledge Infrastructure, WanFang, and SinoMed) and other sources (bioRxiv and reference lists of relevant articles and reviews) were systematically searched for studies that compared regional CTh differences between patients with migraine and healthy controls (HCs) up to May 15, 2020. A CBMA was performed using the Seed-based d Mapping with Permutation of Subject Images approach. In total, we identified 16 studies with 17 datasets reported that were eligible for the CBMA. The 17 datasets included 872 patients with migraine (average sample size 51.3, mean age 39.6 years, 721 females) and 949 HCs (average sample size 59.3, mean age 44.2 years, 680 females). The CBMA detected no statistically significant consistency of CTh alterations in patients with migraine relative to HCs. Sensitivity analysis and subgroup analysis verified this result to be robust. Metaregression analyses revealed that this CBMA result was not confounded by age, gender, aura, attack frequency per month, and illness duration. Our CBMA adds to the evidence of the replication crisis in neuroimaging research that is increasingly recognized. Many potential confounders, such as underpowered sample size, heterogeneous patient selection criteria, and differences in imaging collection and methodology, may contribute to the inconsistencies of CTh alterations in migraine, which merit attention before planning future research on this topic.

## Introduction

Migraine is a highly prevalent neurological condition that affects ~1 billion people worldwide at all ages and more common in women than in men (Feigin et al., 2019). Migraine ranks second in terms of year lived with disability among neurological disorders, leading to major individual and societal burdens (Saylor and Steiner, 2018). Migraine is multifactorial and is often comorbid with other disorders (Bergman-Bock, 2018; Chen et al., 2019). The pathophysiology underlying migraine is complex and remains to be elucidated. It has been widely accepted that trigeminovascular system plays a fundamental role in migraine (Ashina et al., 2019); however, more recent studies have suggested multiple neural networks that comprise brainstem, diencephalic, and cortical structures (Akerman et al., 2017; Puledda et al., 2017; Qubty and Patniyot, 2020).

Cortical thickness (CTh) via surface-based morphometry (SBM) analysis is one of the advanced non-invasive neuroimaging metrics that characterize brain morphometry (Fischl and Dale, 2000). Many studies have been performed to investigate CTh abnormalities in migraine. Brain CTh abnormalities in migraine were found to be associated with age (Chong et al., 2014; Woldeamanuel et al., 2019), gender (Maleki et al., 2012), disease duration (Hubbard et al., 2014; Kim et al., 2014; Magon et al., 2019; Woldeamanuel et al., 2019), attack frequency (Hubbard et al., 2014; Kim et al., 2014; Magon et al., 2019), pain intensity (Hubbard et al., 2014), aura (Messina et al., 2013; Petrusic et al., 2018; Magon et al., 2019), and photosensitivity (Chong et al., 2016). Brain CTh combining cortical surface area and regional volumes have been shown to be highly accurate in distinguishing individual people with chronic migraine from those with episodic migraine and HCs (Schwedt et al., 2015). These studies have helped us to better understand the pathophysiology of migraine (Russo et al., 2018; Ellingson et al., 2019). However, the results from these studies were not consistent and even conflicting. Increased CTh in patients with migraine relative to HCs was observed in the left middle frontal sulcus (Messina et al., 2013), left temporo-occipital incisure (Messina et al., 2013), lateral occipital-temporal cortex (Zhang et al., 2017), and left occipital lobe (Gaist et al., 2018). In contrast, patients with migraine compared to HCs showed reduced CTh in the left superior frontal sulcus (Messina et al., 2013), left middle frontal gyrus (Kim et al., 2014; Magon et al., 2019), left precentral sulcus (Messina et al., 2013), bilateral central sulcus (Magon et al., 2019), bilateral postcentral gyri (Kim et al., 2014), right occipitotemporal area (Magon et al., 2019), left primary visual cortex (Magon et al., 2019), left secondary visual cortex (Magon et al., 2019), left anterior midcingulate (Hubbard et al., 2014), and insula (Maleki et al., 2015; Zhang et al., 2017), while several other studies did not detect any CTh differences between patients with migraine and HCs (Datta et al., 2011; Hougaard et al., 2016; Husøy et al., 2019; Woldeamanuel et al., 2019; Masson et al., 2020). These divergent results hinder us to obtain a clear picture of brain morphometry regarding CTh alterations in migraine.

Coordinate-based meta-analysis (CBMA) is a promising technique to quantitatively pool individual neuroimaging studies to find brain areas consistently involved in particular neuropsychiatric disorders across studies (Muller et al., 2018; Tahmasian et al., 2019). Recently, CBMA of voxel-based morphometry (VBM) studies showed no consistency of gray matter (GM) volume/density alterations in migraine relative to HCs (Masson et al., 2020; Sheng et al., 2020b; Wang et al., 2020). CTh has been shown to be more sensitive than VBM to characterize brain morphometry (Hutton et al., 2009; Lai et al., 2020). To date, no CBMA of CTh studies in migraine has ever been reported. With the development of the algorithm (Albajes-Eizagirre et al., 2019a,c), CBMA has been recently applied to quantify CTh alterations in major depressive disorder (Li et al., 2020). Therefore, we aimed to conduct a CBMA of studies that investigate CTh differences at the whole-brain cortical level between patients with migraine and HC subjects using the Seed-based d Mapping with Permutation of Subject Images (SDM-PSI) approach (Albajes-Eizagirre et al., 2019a,c).

## Materials and Methods

This study conformed to the Preferred Reporting Items for Systematic Reviews and Meta-Analyses (PRISMA) checklist (Moher et al., 2009) and followed guidelines and the recent recommendations for neuroimaging meta-analysis (Muller et al., 2018; Tahmasian et al., 2019). The protocol (CRD42020175789) was registered in the International Prospective Register of Systematic Reviews (PROSPERO).

### Literature Search

We systematically and comprehensively searched the online electronic databases PubMed (https://pubmed.ncbi.nlm.nih.gov/), EMBASE (https://www.embase.com/), and Web of Science (http://apps.webofknowledge.com/) on March 16, 2020, for records published in English, using the following keywords: “migraine” and (“cortical thickness” or “cortical thinning” or “surface-based morphometry”). The searches were updated on May 15, 2020. We also searched China National Knowledge Infrastructure (CNKI, https://www.cnki.net/), WanFang (www.wanfangdata.com.cn), and SinoMed (http://www.sinomed.ac.cn/) for studies published in Chinese. No restrictions were incorporated in the search itself. Additionally, the reference lists of the included articles and any relevant review articles were manually reviewed for other potentially qualified studies. We also searched bioRxiv (https://www.biorxiv.org/about-biorxiv) for unpublished preprints that were qualified for the meta-analysis.

### Study Selection

To be included, the study needed to satisfy the following inclusion criteria: (1) enrollment of adult patients with migraine according to the accepted criteria; (2) case-control studies that employed a whole-brain cortical analysis to compare regional CTh differences between patients with migraine and HCs; (3) studies with significant CTh results that reported three-dimensional peak coordinates in standard Montreal Neurological Institute (MNI) or Talairach space; and (4) studies with non-significant CTh results. Exclusion criteria were as follows: (1) there were seven or fewer participants in either the migraine group or the HC group (Tahmasian et al., 2019); (2) the study did not list three-dimensional coordinates of significant results regarding regional CTh differences between patients with migraine and HCs; (3) the study only performed region-of-interest analysis or global CTh analysis; (4) the study lacked a direct migraine-HC group comparison of regional CTh difference; (5) the patient sample was duplicated or overlapped with another study with a larger sample size; (6) no baseline comparison results were reported in case of a longitudinal study; and (7) the publications were reviews, study protocols, conference abstracts, correspondence, and editorials.

### Data Extraction

The following information was extracted from the retrieved studies: the first author's name, year of publication, sample size, age, sex distribution, patient type (episodic/chronic migraine), the number of patients with and without aura, illness duration, attack frequency per month, magnetic resonance imaging (MRI) scanner manufacturer and platform, field strength, head coil, MRI sequence, repetition time (TR)/echo time (TE), voxel size, imaging processing software package, smooth kernel, statistical model, covariate, statistical threshold, peak coordinates, the height of the peaks (*t*-values, *z*-values, or *p*-values. The latter two values could be converted to *t*-values via the web utilities: https://www.sdmproject.com/utilities/?show=Statistics), and their stereotactic reference space (MNI or Talairach).

### Quality Assessment

Currently, no official tools have been established to assess the quality of CTh studies. A 12-point checklist (Supplementary Table 1), which was based on a previous CTh meta-analysis (Li et al., 2020), was utilized for the quality assessment of the included studies. This checklist constitutes a 4-point scale for evaluation of sample characteristics (0–4 points), a 5-point scale for assessment of methods for image acquisition and analysis (0–5 points), and a 3-point (0–3 points) scale for assessment of results and conclusions. Studies recording an overall score of ≥ 10 were considered as good quality, studies with an overall score between 7 and 9 as moderate quality, and an overall ≤ 7 as poor quality.

### Data Analysis

#### Main CBMA by Pooling All Included Studies

Main CBMA of all included studies was performed using SDM-PSI (version 6.21, www.sdmproject.com) as described in detail previously (Albajes-Eizagirre et al., 2019a,c). The standard SDM-PSI pipeline was followed for the CMBA and is as follows: (1) preprocessing was first conducted to calculate an image of the lower bound and an image of the upper bound of possible effect sizes for each study separately using a specific cortical GM FreeSurfer mask (Li et al., 2020) for meta-analyzing the SBM studies, a 20-mm full-width half-maximum anisotropic Gaussian kernel (Radua et al., 2014), and a 2-mm voxel size; (2) mean analysis was then performed to estimate the Hedge-corrected effect sizes in a standard random-effects model using MetaNSUE algorithms (Radua et al., 2015; Albajes-Eizagirre et al., 2019b), multiple imputations of maximum likelihood estimation, and Rubin's rules (Li et al., 1991); (3) finally, voxel-wise results are determined using threshold-free cluster enhancement family-wise error rate (TFCE-FWER) *p* < 0.05 corrected for multiple comparisons and extent threshold of ≥ 10 voxels. This statistical thresholding has been suggested to be neither too conservative nor too liberal in the simulation work (Albajes-Eizagirre et al., 2019a,c).

### Sensitivity Analysis

To assess the stability of the results identified in the main CBMA, a sensitivity analysis was performed by repeating the same analyses by consecutively removing one study at a time.

### Heterogeneity Analysis

Heterogeneity of significant brain clusters identified in the main CBMA was estimated using the *I*^2^ statistic, and *I*^2^ > 50% was defined as high heterogeneity across studies (Albajes-Eizagirre et al., 2019c).

### Publication Bias Analysis

The risk of publication bias was evaluated using the Egger test (Egger et al., 1997) by extraction of the values from the significant peaks in the main CBMA. A threshold at *p* < 0.05 was set of significance.

### Subgroup Meta-Analysis

Subgroup meta-analyses were performed to investigate the possible effects of the results on the overall conclusions if at least 10 datasets were available based on (1) migraine patients with aura/without aura, (2) patients with episodic migraine/chronic migraine, and (3) use of using 3.0 T/1.5-T MRI scanners.

### Metaregression Analysis

We conducted random-effects metaregression analyses, exploring if regional CTh alterations across studies might be moderated by main study characteristics, including age, gender, aura, attack frequency per month, and illness duration if relevant information was available from at least 10 datasets. A statistical threshold was set at *p* < 0.05 (TFCE-FWER) and a cluster extent of 10 voxels.

## Results

### Study Selection

**Figure 1:**
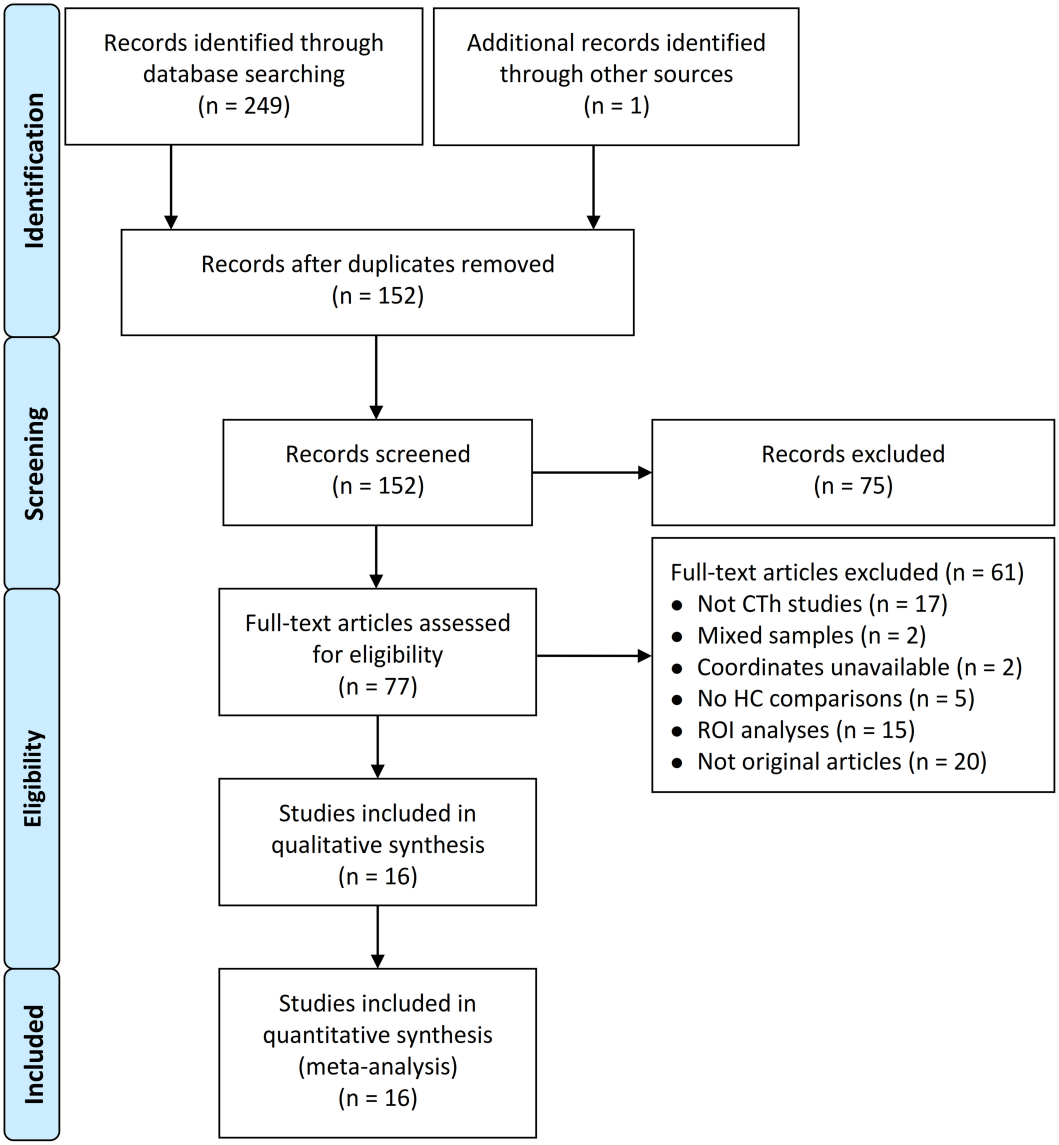
Flowchart of study selection process. CTh, cortical thickness; HC, healthy control; ROI, region of interest.

After deleting the repetitive publications from the electronic database and manual searches, 250 records were screened. Based on the eligibility criteria, a total of 16 studies that reported 17 datasets were finally included in the CBMA (Datta et al., 2011; Maleki et al., 2012, 2015; Messina et al., 2013; Chong et al., 2014; Hubbard et al., 2014; Kim et al., 2014; Hougaard et al., 2016; Zhang et al., 2017; Gaist et al., 2018; Petrusic et al., 2018; Husøy et al., 2019; Magon et al., 2019; Woldeamanuel et al., 2019; Lai et al., 2020; Masson et al., 2020). Figure 1 presents the PRISMA flowchart.

### Study Characteristics

Publication year of the included studies ranged from 2011 to 2020. The 17 datasets included 872 patients with migraine [average sample size, 51.3 [range, 11–131]; mean age, 39.6 years [range, 30.8–57.4 years]; 721 females] and 949 HC subjects [average sample size 59.3 [range, 11–309]; mean age, 44.2 years [range, 29.1–58.7years]; 680 females]. There were no statistically significant differences between patients with migraine and HC subjects in average sample size (*p* = 0.76), mean age [standardized mean difference = 0.018; 95% confidence interval [CI] = −0.078 to 0.11, *z* = 0.37, *p* = 0.71], or gender distribution [relative risk = 1.02, 95% CI = 0.95 to 1.1, *z* = 0.54, *p* = 0.59]. Of the 17 datasets, 10 evaluated patients with episodic migraine, 2 evaluated patients with chronic migraine, 2 evaluated patients with episodic and chronic migraine, and the remaining 3 did not explicitly indicate the patient type. The information regarding migraine patients with aura or without aura from 13 datasets, illness duration from 14 datasets, and attack frequency per month from 12 datasets were available. MRI data were acquired mostly on 3.0-T machines (15 of the 17 datasets) and 1.5-T machines (2 of the 17 datasets). Fourteen of 17 datasets used FreeSurfer software packages, and 3 used Computational Anatomy Toolbox (CAT) to analyze regional CTh differences between patients and HC subjects. The demographic and clinical characteristics and imaging characteristics are listed in Tables 1, 2, respectively. The scores of quality assessment are shown in Table 1. Overall, all included studies reached a score of either “good” or “moderate.”

**Table 1:** Demographic and clinical characteristics of CTh studies included in the meta-analysis.

**Study**	**Migraine type**	**Sample (female)**	**WoA/WA**	**Age (years, SD)**	**Duration (years, SD)**	**Attack frequency/month (SD)**	**Medication**	Quality score^*^
Datta et al., 2011	EM	Patients 28 (24)^a^	0/28	35 (6)	19.1 (NA)	4.1 (5.6)	NA	11
		Patients 28 (24)^b^	28/0	35 (7)	15 (NA)	3.4 (3.8)		11
		Controls 28 (24)		33 (6)				
Maleki et al., 2012	EM	Patients 11 (0)	NA/NA	42.7 (9.3)	≥3 years	NA	Medicated	10
		Controls 11 (0)		43 (9.9)				
Messina et al., 2013	NA	Patients 63 (42)	31/32	37.2 (NA)	17 (NA)	<1/month, 8 patients	19 Medicated	11
		Controls 18 (13)		36.9 (NA)		1–3/month, 32 patients		
						>3/month, 23 patients		
Chong et al., 2014	EM	Patients 27 (22)	18/9	33.6 (12.3)	16 (9.2)	6.4 (3.0)	Medication-free	11.5
		Controls 32 (25)		35.3 (11.6)				
Hubbard et al., 2014	EM/CM	Patients 17 (13)	NA/NA	41.71 (12.2)	12.53 (8.41)	11.65 (10.07)	14 Medicated	10.5
		Controls 18 (14)		38.89 (11.25)				
Kim et al., 2014	EM/CM	Patients 56 (56)	56/0	35.7 (9.5)	10.9 (5.8)	10.1 (5.7)	Medicated	11
		Controls 34 (34)		34.2 (9.3)				
Maleki et al., 2015	EM	Patients 46 (46)	46/0	34.7 (10.4)	15.6 (9.5)	<2/month, 13 patients	Majority medicated	11.5
		Controls 46 (46)		34.1 (10.6)		2–6/month, 17 patients		
						>8/month, 16 patients		
Hougaard et al., 2016	EM	Patients 60 (42)	0/60	33.36 (NA)	NA	1	Medication-free?	11
		Controls 60 (42)		33.39 (NA)				
Zhang et al., 2017	EM	Patients 32 (24)	32/0	38.3 (10.16)	9.5 (6.23)	3.36 (2.55)	Medication-free	11
		Controls 32 (24)		38.8 (10.02)				
Gaist et al., 2018	NA	Patients 166 (166)	0/166	48.0 (6.6)	14.96 (NA)	NA	NA	10.5
		Controls 137 (137)		48.0 (7.7)				
Petrusic et al., 2018	EM	Patients 48 (36)	0/48	39.3 (11.2)	18.5 (10.5)	0.68 (0.93)	Medication-free	11
		Controls 30 (23)		39.6 (12.0)				
Husøy et al., 2019	NA	Patients 80 (60)	NA	57.4 (4.3)	NA	NA	NA	10.5
		Controls 309 (124)		58.7 (4.1)				
Magon et al., 2019	EM	Patients 131 (109)	93/38	30.8 (9.0)	14.1 (8.5)	3.3 (2.5)	3 Medicated	11.5
		Controls 115 (81)		29.1 (7.2)				
Woldeamanuel et al., 2019	CM	Patients 30 (24)	17/13	40 (14)	26 (13)	27 (12)	Medicated	11
		Controls 30 (24)		40 (14)				
Masson et al., 2020	EM	Patients 19 (13)	0/19	33.6 (11.5)	16.8 (7.4)	3.3 (1.1)	Medication-free?	10.5
		Controls 19 (13)		32.7 (8.7)				
Lai et al., 2020	CM	Patients 30 (23)	NA/NA	33.2 (9.8)	13.2 (8.8)	24.0 (5.3)	Medicated	11
		Controls 30 (22)		32.4 (8.3)				

*CTh, cortical thickness; EM, episodic Migraine; CM, chronic migraine; WoA, patients with migraine without aura; WA, patients with migraine with aura; SD, standard deviation; controls, headache-free controls; NA, not available*;

a *migraine with aura*;

b *migraine without aura*.

* *Twelve points in total*.

**Table 2:** Imaging characteristics of the CTh studies included in the meta-analysis.

**Study**	**MRI scanner**	**Field strength**	**Head coil**	**MRI sequence**	**TR/TE (mm/mm)**	**Voxel size (mm^**3**^)**	**Software**	**FWHM (mm)**	**Analytic model**	**Covariate**	**Threshold**	**Quality control**
Datta et al., 2011	Trio, Siemens	3.0 T	8-Channel	MPRAGE	1,620/3.09	1 × 1 × 1	FreeSurfer	10	Random-effects models/*t*-test	Age and gender	*P* < 0.05 (FDR)	NA
Maleki et al., 2012	Trio, Siemens	3.0 T	8-Channel	MPRAGE	2,100/2.74	1.33 × 1.0 × 1.0	FreeSurfer	10	Vertex-wise GLM	NA	*P* < 0.05 (MCS)	NA
Messina et al., 2013	Intera, Philips	3.0 T	NA	FFE	25/4.6	0.89 × 0.89 × 0.8	FreeSurfer v4.5	10	Vertex-wise GLM	Age, gender, whole-hemisphere average cortical thickness and cortical surface area	*P* < 0.01 (FDR)	NA
Chong et al., 2014	Trio, Siemens	3.0 T	12-Channel	MPRAGE	2,400/3.16	1 × 1 × 1	FreeSurfer v5.3	15	Vertex-wise GLM	Depression, anxiety, and migraine burden	*P* < 0.025 (MCS)	Yes
Hubbard et al., 2014	Trio, Siemens	3.0 T	12-Channel	MPRAGE	2,500/3.44	0.9 × 0.9 × 1	FreeSurfer v5.3	10	Vertex-wise GLM	Age	*P* < 0.05 (RFT)	Yes
Kim et al., 2014	Trio, Siemens	3.0 T	12-Channel	MPRAGE	1,780/2.34	1 × 1 × 1	FreeSurfer v5.1	15	Vertex-wise GLM	Age	*P* < 0.05 (MCS)	NA
Maleki et al., 2015	Siemens	3.0 T	NA	MPRAGE	2,100/2.74	1.33 × 1.0 × 1.0	FreeSurfer	5	Vertex-wise GLM	Age, and TIV	*P* < 0.05 (MCS)	NA
Hougaard et al., 2016	Intera, Philips	3.0 T	32-Channel	TFE	9,900/4.6	1 × 1 × 1	FreeSurfer	10	Vertex-wise GLM	Age, gender, disease duration, and attack frequency	*P* < 0.05 (PBNPA)	NA
Zhang et al., 2017	Trio, Siemens	3.0 T	12-Channel	MPRAGE	2,530/2.34	1 × 1 × 1	CAT12	15	Voxel-wise *t*-test	NA	*P* < 0.05 (FDR)	NA
Gaist et al., 2018	Verio, Siemens	3.0 T	32-Channel	FLASH	18.7/2.2	NA	FreeSurfer v6.0.0	5	Vertex-wise GLM	AGE	*P* < 0.05 (MCS)	NA
Petrusic et al., 2018	Signa, GE	1.5 T	8-Channel	FSPGR	8.12/3.6	0.47 × 0.47 × 1.4	FreeSurfer v5.3	10	Vertex-wise GLM	Age and gender	*P* < 0.05 (MCS)	NA
Husøy et al., 2019	Signa, GE	1.5 T	8-Channel	MPRAGE	10.2/4.1	1.2 (slice thickness)	FreeSurfer v5.3	10	Vertex-wise GLM	Age and gender	*P* < 0.05 (FDR)	NA
Magon et al., 2019	Trio, Siemens; Signa, GE; Achieva, Philips	3.0 T	8- or 12-Channel	NA	3.99/9,000, 2.98/2,300, 4.6/9,900, 1.5/6,300, 2.98/2,300	1 × 1 × 1	FreeSurfer v5.3	NA	Vertex-wise ANCOVA model	Age, gender and MRI scanner	*P* < 0.05 (FDR)	Yes
Woldeamanuel et al., 2019	Discovery, GE	3.0 T	8-Channel	IR-FSPGR	5.9/2	0.9 × 0.9 × 1	FreeSurfer v5.3.0	NA	Vertex-wise GLM	Age	*P* < 0.001 (FDR)	NA
Masson et al., 2020	Prisma, Siemens	3.0 T	64-Channel	MPRAGE	3,500/2.25	0.9 × 0.9 × 0.9	CAT12	15	Voxel-wise *t*-test	Age and gender	*P* < 0.05 (TFCE, FWE)	NA
Lai et al., 2020	Trio, Siemens	3.0 T	32-Channel	MPRAGE	2,530/3.03	1 × 1 × 1	CAT12	20	Voxel-wise *t*-test	Age and gender	*P* < 0.05 (FDR)	NA

*CTh, cortical thickness; MRI, magnetic resonance imaging; TR/TE, repetition time/echo time; FWHM, full-width half-maximum; MPRAGE, magnetization prepared rapid gradient echo; FDR, false discovery rate; GLM, general linear model; MCS, Monte Carlo Simulation; NA, not available; FFE, fast field echo; TIV, total intracranial volume; RFT, random field theory; TFE, turbo field echo; PBNPA, Permutation-Based Non-Parametric Analysis; CAT, Computational Anatomy Toolbox; FLASH, fast low angle shot; FSPGR, fast spoiled gradient recalled echo; IR-FSPGR, inversion recovery prepared fast spoiled gradient recalled sequence; TFCE, threshold-free cluster enhancement*.

### Main CBMA

The main CBMA of all included datasets showed no significant brain clusters of regional CTh difference between patients with migraine and HC subjects (TFCE-FWER corrected *p* < 0.05 and voxel extent ≥ 0).

### Sensitivity Analysis

The sensitivity analysis revealed that the result of no consistent difference in regional CTh between patients with migraine and HC subjects remained in all combinations of datasets.

### Heterogeneity Analysis and Publication Bias Analysis

The lack of significant brain clusters identified in the main CBMA prevented us from performing heterogeneity analysis and publication bias analysis.

### Subgroup Meta-Analysis

Subgroup meta-analysis of datasets in patients with episodic migraine (*n* = 10), of datasets using 3.0-T MRI scanners (*n* = 15), and of datasets using FreeSurfer software packages (*n* = 14) demonstrated no significant findings (TFCE-FWER corrected *p* < 0.05 and voxel extent ≥ 10). Other subgroup meta-analyses were not performed because of the insufficient datasets included.

### Metaregression Analysis

Metaregression analyses revealed that age, gender, aura, attack frequency per month, and illness duration were not moderators that influence the CBMA result of regional CTh difference across studies (TFCE-FWER corrected *p* < 0.05 and voxel extent ≥ 10).

## Discussion

To the best of our knowledge, this is the first CBMA of CTh studies in migraine. Using the SDM-PSI meta-analytical approach, our CBMA that included 17 datasets comprising 872 patients and 949 controls detected no statistically significant consistency of CTh alterations in patients with migraine relative to HCs. This lack of specific CTh alterations indicates that CTh analysis is not a reliable and reproducible metric as a potential biomarker of migraine. Although little is known about the exact reasons for the absence of consistency of CTh alterations in migraine, we will discuss the possible sources and factors from the variability of sample size and heterogeneous patient selection criteria to imaging collection and methodological differences across independent studies.

There is an increasing concern regarding the reliability and reproducibility in neuroimaging research (Nichols et al., 2017). A small sample size with low statistical power undermines the reliability of neuroscience (Button et al., 2013). A power null result from CTh analysis mainly depended upon the thickness difference to be detected, the applied level of surface-based smoothing, and the type I error rate (Pardoe et al., 2013). Calculations to estimate the appropriate sample size should be undertaken before the study has begun (Pardoe et al., 2013). A well-powered cross-sectional CTh study required ~50 subjects per group to detect a 0.25-mm CTh difference (Pardoe et al., 2013). Of the 17 datasets included in the CBMA, the sample sizes range from 11 to 166 (mean, 51.3) in the patient groups and from 11 to 309 (mean, 59.3) in the HC groups, of which the majority (*n* = 13) enrolled participants with small sample size of fewer than 50 subjects per group. Only three studies included in the CBMA conducted prior statistical power calculations with different sample sizes required (Datta et al., 2011; Husøy et al., 2019; Masson et al., 2020). These studies with small sample sizes have a higher probability of false-positive results that affected the generalizability of the obtained results. Although it is challenging in practice, data sharing or multicenter collaboration to increase the sample size (and therefore power) is highly needed (Button et al., 2013; Nichols et al., 2017).

Heterogeneous patient selection criteria make it difficult to define consistent migraine characteristic alterations of CTh. Migraine is a heterogeneous neurological disease. Some datasets enrolled only episodic migraineurs (Datta et al., 2011; Maleki et al., 2012, 2015; Hougaard et al., 2016; Zhang et al., 2017; Petrusic et al., 2018; Magon et al., 2019; Masson et al., 2020), whereas some other datasets included both episodic and chronic migraineurs (Hubbard et al., 2014; Kim et al., 2014) or only chronic migraineurs (Woldeamanuel et al., 2019; Lai et al., 2020). Majority of the datasets (*n* = 14) in the CBMA included patients with mixed gender (Datta et al., 2011; Messina et al., 2013; Chong et al., 2014; Hubbard et al., 2014; Kim et al., 2014; Hougaard et al., 2016; Zhang et al., 2017; Petrusic et al., 2018; Husøy et al., 2019; Magon et al., 2019; Woldeamanuel et al., 2019; Lai et al., 2020; Masson et al., 2020), whereas two datasets included only female migraine patients (Maleki et al., 2015; Gaist et al., 2018), and one dataset only male migraine patients (Maleki et al., 2012). Some datasets included only patients with aura (Datta et al., 2011; Hougaard et al., 2016; Gaist et al., 2018; Petrusic et al., 2018) or only those without aura (Datta et al., 2011; Kim et al., 2014; Maleki et al., 2015; Zhang et al., 2017; Masson et al., 2020), whereas some other studies included both patients with migraine without aura and those with aura (Messina et al., 2013; Chong et al., 2014; Magon et al., 2019; Woldeamanuel et al., 2019). Individual CTh studies showed that the observed regional pattern of CTh abnormalities in patients with migraine was influenced by age (Chong et al., 2014; Woldeamanuel et al., 2019), gender (Maleki et al., 2012), disease duration (Hubbard et al., 2014; Kim et al., 2014; Magon et al., 2019; Woldeamanuel et al., 2019), attack frequency (Hubbard et al., 2014; Kim et al., 2014; Magon et al., 2019; Lai et al., 2020), pain intensity (Hubbard et al., 2014), the presence of aura (Messina et al., 2013; Petrusic et al., 2018; Magon et al., 2019), and photosensitivity (Chong et al., 2016). Of note, our subgroup CBMA of CTh datasets in patients with episodic migraine (*n* = 10) did not find any significant result as well. Other subgroup meta-analyses could not be conducted because of insufficient datasets included (< 10). Whether the lack of significant effect was specific to the patient subpopulation, more homogeneous studies (clinical subtypes) for inclusion in the subgroup CBMA are warranted to investigate this potential effect in future studies. In addition, two datasets were cross-sectional population-based studies (Gaist et al., 2018; Husøy et al., 2019), and the rest are clinic-based studies that the former minimized the selection biases compared to the latter. Migraine is a recurrent headache disorder characterized by a cycle of attacks including pain-attack ictal and pain-free interictal phases. Different patterns of morphometric GM changes detected via VBM and dynamic variations in the anatomical microstructure of the thalamus detected via diffusion tensor imaging between ictal and interictal phases were observed in migraine, which suggests that abnormal structural plasticity may be an important mechanism of migraine pathology (Coppola et al., 2014, 2015). However, no CTh studies to date have been conducted to explore headache phase-related cortical plasticity in migraine. An extensive literature has shown that a wide range of psychiatric disorders, especially anxiety and depression, can accompany migraine (Minen et al., 2016; Seng and Seng, 2016; Bergman-Bock, 2018; Korkmaz et al., 2019). Previous studies revealed cortical abnormalities in depression (Schmaal et al., 2017; Suh et al., 2019; Li et al., 2020) and anxiety disorders (Zhao et al., 2017; Molent et al., 2018; Carnevali et al., 2019; Suffren et al., 2019). However, these psychiatric problems are often underdiagnosed and have not been thoroughly assessed in CTh studies in migraine. Only a few studies in the CBMA included patients at the medication-free state (Chong et al., 2014; Zhang et al., 2017; Petrusic et al., 2018). Medication status and type are other potential confounders that may influence CTh findings in migraine; however, no CTh studies have attempted to evaluate such effects.

Differences in imaging collection and methodology of CTh analyses may also have contributed to the absence of consistency from CTh studies in migraine. Previous reports showed that results of CTh analyses can be influenced by scanner platform (Yang et al., 2016; Fortin et al., 2018), field strength (Han et al., 2006; Park et al., 2008; Lusebrink et al., 2013), pulse sequence (Han et al., 2006; Wonderlick et al., 2009; Yan et al., 2020), the number of coil channels (Yan et al., 2020), scanner relocation (Melzer et al., 2020), and imaging sites (Iscan et al., 2015; Fortin et al., 2018). As shown in Table 2, differences in scanner manufacturer and platform (Siemens, Philips, and GE), field strength (3.0 and 1.5 T), head coil (8-, 12-, 32-, and 64-channel), MR sequence (magnetization prepared rapid gradient echo, fast field echo, turbo field echo, fast low angle shot, and fast spoiled gradient recalled echo sequence), TR/TE, and voxel size (from 1.33 × 1.0 × 1.0 to 0.89 × 0.89 × 0.8 mm^3^) across studies were noted. Besides, variations in computing workstation types (Gronenschild et al., 2012), operating systems (Gronenschild et al., 2012; Glatard et al., 2015), processing pipelines and software packages (Gronenschild et al., 2012; Righart et al., 2017; Seiger et al., 2018), the extent of smoothing (Bernal-Rusiel et al., 2010), and statistical strategies (Messina et al., 2013; Chong et al., 2014; Zhang et al., 2017; Petrusic et al., 2018) may also have produced inconsistent results. Most individual studies did not explicitly state the computing workstation types and operating systems used in the CTh analyses. The CTh studies in migraine included in the CBMA used divergent processing pipelines and software packages (different versions of FreeSurfer and CAT12), smoothing kernels, and statistical strategies. Specially, four studies revealed that the use of a more liberal uncorrected threshold produced more positive results (Messina et al., 2013; Chong et al., 2014; Zhang et al., 2017; Petrusic et al., 2018). Moreover, there is increasing awareness that image quality can systematically bias the results (Reuter et al., 2015; Ducharme et al., 2016; Canna et al., 2018). However, only three of the studies included in the CBMA explicitly conducted a visual inspection and manual correction of topological errors for quality control (Chong et al., 2014; Hubbard et al., 2014; Magon et al., 2019). These differences make direct comparisons between the different studies difficult. In order to improve the reproducibility of the results, higher field strength, multiecho sequence, more coil channels, harmonization of CTh measurements across scanners and sites, use of homogeneous sets of platforms, constant operating systems, and quality control are recommended in future studies.

As discussed above, many potential confounders may contribute to the inconsistencies of CTh alterations in migraine, which merit attention in future studies. Of the 17 CTh datasets included in the CBMA, 9 reported null finding in patients with migraine relative to HCs using corrected thresholds for multiple comparisons (Datta et al., 2011; Maleki et al., 2012; Chong et al., 2014; Hougaard et al., 2016; Petrusic et al., 2018; Husøy et al., 2019; Woldeamanuel et al., 2019; Masson et al., 2020). In contrast, a multicenter study from four academic headache centers showed a significantly thinner CTh in 131 patients with migraine compared with 115 HC subjects and further demonstrated CTh differences between patients with migraine with and without aura (Magon et al., 2019). The meaningful significant results from this study seem more reliable than other single studies with small sample sizes because they were from a large cohort of patients and were corrected for multiple comparisons controlling for age, gender, and MRI scanner (Magon et al., 2019). However, the quantitative CBMA of these studies detected no significantly consistent CTh alterations in migraine. Is migraine truly not associated with CTh alterations? Are significant CTh alterations observed in the studies secondary, or specified to migraine subgroups, or just a reflection of structural plasticity of the migraine cycle? To answer these questions and to obtain reliable results, we need to design longitudinal population-based studies at different migraine phases that (1) recruit subgroup-homogeneous patients with appropriate sample size; (2) use standardized imaging collection protocols with high field strength, multiecho sequence, and a high number of coil channels; (3) harmonize CTh measurements across scanners and sites with homogeneous sets of platforms and constant operating systems; (4) perform quality control; and (5) apply latest well-validated processing and analysis pipelines with correction for multiple comparisons controlling for the age, gender, comorbidities, and medication. Besides, longitudinal multimodal neuroimaging studies would contribute to elucidate whether CTh alterations are secondary to chronic functional abnormalities.

Several limitations to our CBMA must be considered. First, given the clinical heterogeneity of migraine and the lack of sufficient original studies, we were unable to conduct separate subgroup CBMA to identify the effects of potential moderators, such as migraine with aura vs. migraine without aura, male migraine vs. female migraine, and episodic migraine vs. chronic migraine. More CTh studies in migraine with homogeneous subtypes are needed to characterize the CTh patterns. Second, the present meta-analysis is coordinate-based rather than image-based or mixed coordinate- and image-based, which may lead to biased results. Future studies with imaging data sharing would be helpful to obtain more accurate results.

## Conclusions

The present CBMA detected no consistent CTh alterations in patients with migraine relative to HCs. Our CBMA adds to the evidence of the replication crisis in neuroimaging research that is increasingly recognized (Muller et al., 2017; Tahmasian et al., 2018). Whether migraine is truly associated with CTh alterations is still argued. Many potential confounders, such as underpowered sample size, heterogeneous patient selection criteria, and differences in imaging collection and methodology, may contribute to the inconsistencies of CTh alterations in migraine, which merit attention before planning future research on this topic. Longitudinal population-based multimodal neuroimaging studies at different migraine phases that subtype homogeneous patients with well-powered sample sizes using standardized imaging collection protocols and well-validated processing and analysis pipelines controlling for age, gender, comorbidities, and medication are required to improve the reliability of the results that characterize CTh alterations in migraine.

## Data Availability Statement

The data analyzed in this study is subject to the following licenses/restrictions: The datasets generated and/or analyzed during the current study are available from the corresponding author on reasonable request. Requests to access these datasets should be directed to PingLei Pan, panpinglei@163.com.

## Author Contributions

JZ, FC, and PP conceived and designed the study. LS, HM, and YS performed the experiments. LS, JZ, and HM analyzed the data. LS, HM, and YS prepared the manuscript. JZ, FC, and PP reviewed and edited the manuscript. All authors read and approved the final manuscript.

## Conflict of Interest

The authors declare that the research was conducted in the absence of any commercial or financial relationships that could be construed as a potential conflict of interest.

## Abbreviations

CAT: Computational Anatomy Toolbox
CBMA: coordinate-based meta-analysis
CNKI: China National Knowledge Infrastructure
CTh: cortical thickness
FWER: family-wise error rate
GM: gray matter
HCs: healthy controls
MNI: Montreal Neurological Institute
MRI: magnetic resonance imaging
PRISMA: Preferred Reporting Items for Systematic Review and Meta-Analysis
SBM: surface-based morphometry
SDM-PSI: Seed-based d Mapping with Permutation of Subject Images
SPGR: spoiled gradient-echo
TFCE: threshold-free cluster enhancement
TR/TE: repetition time/echo time
VBM: voxel-based morphometry.
