# Supplementary material for: Cortical Thickness in Migraine: A Coordinate-Based Meta-Analysis
Source: Front Neurosci. 2021 Jan 6;14:600423. doi: 10.3389/fnins.2020.600423 (PMC7815689; doi:10.3389/fnins.2020.600423)
Supplement: Supplementary file 1 [file Table_1.DOCX]

**Table S1. The checklist of quality assessment for the included cortical thickness studies**

| **12-point checklist** |
| --- |
| **Category 1: Subjects** |
| 1. Patients were evaluated prospectively, specific diagnostic criteria were applied, and demographic data were reported. |
| 2. Healthy comparison participants were evaluated prospectively; psychiatric and medical illnesses were excluded. |
| 3. Important variables (e.g., age, gender, illness duration, migraine type, attack frequency, medication) were checked either via stratification or statistics. |
| 4. Sample size per group: ≥ 20, scores 1; ≥ 7, scores 0.5 |
| **Category 2: Methods for image acquisition and analysis** |
| 5. Magnet strength: 3T, scores 1; 1.5T, scores 0.5 |
| 6. Quality control is performed. |
| 7. The imaging technique used was clearly described so that it could be reproduced. |
| 8. Whole brain cortical analysis was automated without a previously defined region. |
| 9. Spatial coordinates were reported in a standard space (e.g., Talairach or MNI coordinates). |
| **Category 3: Results and conclusions** |
| 10. Information about the covariates used, such as age and gender in the statistical model were provided. |
| 11. Statistical results were corrected for multiple comparison scores 1, uncorrected scores 0.5. |
| 12. Conclusions were consistent with the results obtained, and the limitations were discussed. |
| **Total score** |
